# Phase-separated protein dynamics are affected by fluorescent tag choice

**DOI:** 10.17912/micropub.biology.000143

**Published:** 2019-08-19

**Authors:** Celja J Uebel, Carolyn M Phillips

**Affiliations:** 1 Department of Biological Sciences, University of Southern California, Los Angeles, California, United States of America

**Figure 1.**
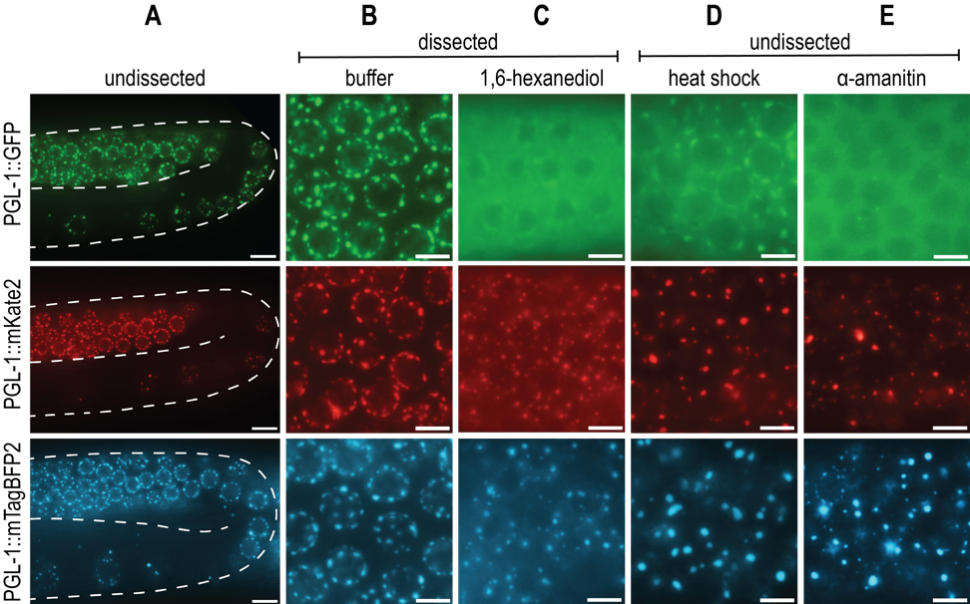
**Figure 1. PGL-1 fluorescently tagged with mKate2 or mTagBFP2 has distinct phase-separation dynamics compared to PGL-1::GFP. (A)** Representative live images of endogenously tagged PGL-1::GFP (top row), PGL-1::mKate2 (middle row), and PGL-1::mTagBFP2 (bottom row) expression in the late pachytene and diplotene region of undissected *C. elegans* gonads. Scale bar, 10µm**. (B)** Live images of endogenously tagged PGL-1 in late pachytene zone of buffer dissected gonad. Scale bars, 5µm. **(C)** Live images of late pachytene region of gonads dissected in 5% 1,6-hexanediol, an aliphatic alcohol. Scale bars, 5µm. **(D)** Live images of undissected late pachytene region immediately after heat shock of 34°C for 3 hours. Scale bars, 5µm**. (E)** Live images of pachytene region 5 hours after microinjection of 200µg/mL of the transcriptional inhibitor, α-amanitin. Scale bars, 5µm.

## Description

Biological liquid-liquid phase separation gives rise to dense protein-protein or protein-RNA condensates that are distinct from the surrounding bulk cytoplasmic or nuclear phase. These condensates, comprised of many multivalent, weak, and hydrophobic interactions, perform a wide variety of physiological functions and are sensitive to changes in the cellular environment (Shin and Brangwynne, 2017). One notable phase-separated condensate is the P granule, a *C. elegans* germline-specific mRNA surveillance center. While the liquid nature of P granules was first described by Brangwynne et al. (2009), additional P granule properties and protein dynamics have been examined with a variety of *in vivo* techniques. The advances in CRISPR/Cas9 gene editing techniques make it possible to endogenously tag PGL-1, a major constituent of P granules, and study protein dynamics *in vivo* via live fluorescent imaging. PGL-1 tagged with Green Fluorescent Protein (GFP) is widely used for the study of P granules, and forms distinct perinuclear germline foci consistent with previous observations of P granules (A, top row) (Pitt et al., 2000; Strome and Wood, 1982).

Due to an interest in visualizing additional germline proteins, we created both *pgl-1::mKate2* (A, middle row) and *pgl-1::mTagBFP2* (A, bottom row) endogenously tagged strains that would allow us to visualize multiple fluorescent proteins with differing excitation and emission spectra. All three C-terminally fluorescently tagged PGL-1 strains behave similarly to one another under both undissected and dissected control conditions (A, B). However, when we tested conditions that probe P granule properties, we were surprised to find that the mKate2 and mTagBFP2 tagged PGL-1 have strikingly different dynamics from the previously described PGL-1::GFP.

First, 1,6-hexanediol is an aliphatic alcohol that is shown to dissolve liquid-like condensates, but not aggregates or solids (Kroschwald et al., 2017). While the mechanism is not entirely understood, 1,6-hexanediol is thought to disrupt hydrophobic interactions that are important for phase-separated condensate integrity. Consistent with Updike et al. (2011), PGL-1::GFP dissolves in 5% 1,6-hexanediol, as demonstrated by lack of perinuclear foci and increased fluorescent signal in the cytoplasm (C, top row). In contrast, although PGL-1::mKate2 and PGL-1::mTagBFP2 have increased cytoplasmic fluorescent signal, clear perinuclear puncta are still visible, indicating a lack of complete dissolution and perhaps a more aggregate-like consistency (C, middle row, bottom row).

Second, liquid phase separation is influenced by changes in temperature. Higher temperatures introduce greater amounts of entropy into the system, allowing for de-mixing of the condensate into the bulk phase (Alberti et al., 2019). PGL-1::GFP condensates in embryos are observed to dissolve at temperature shifts to 34°C for 1 minute (Putnam et al., 2019). In adult germlines, evidence of P granule de-mixing can be seen by 3 hours at 34°C, where PGL-1::GFP foci are faint and fluorescent signal is heavily cytoplasmic (D, top row). However, this same temperature shift results in large, bright, abnormal foci and low cytoplasmic signal in both PGL-1::mKate2 and PGL-1::mTagBFP2 (D, middle row, bottom row). The majority of these foci appear detached from the nuclear periphery and large, round, abnormal aggregates are also observed in the syncytial gonad rachis (not shown).

Lastly, P granules are protein-RNA condensates, and rely on RNA for their formation. Consistent with observations by Sheth et al. (2010), PGL-1::GFP foci in the pachytene are disrupted 5 hours post-microinjection of α-amanitin, a potent transcriptional inhibitor (E, top row). PGL-1::mKate2 and PGL-1::mTagBFP2 both fail to dissolve, and instead form bright puncta that are abnormal compared to control foci (E, middle row, bottom row).

Both mKate2 and mTagBFP2 are bright, monomeric fluorescent tags derived from TagRFP and are similar in size to GFP (Shcherbo et al., 2009; Subach et al., 2011). Here we show that the *in vivo* dynamics of PGL-1 foci are drastically altered when tagged with mKate2 or mTagBFP2 and appear to be more solid or aggregate-like than when tagged with GFP. While the mechanism behind this effect is unclear, it is possible that specific tag-to-tag or tag-to-protein interactions are altering the phase dynamics. Additionally, the high expression levels of PGL-1 may make it more prone to aggregation. Ultimately, our data shows fluorescent tag choice is sufficient to perturb phase-separation dynamics, and we recommend ensuring that the dynamics of new fluorescently tagged proteins are consistent with previous literature, *in vitro* experiments, untagged protein, or additional fluorescent tags.

## Methods

**Strain Construction:** The *C. elegans* wild-type N2 (Bristol) was used as the injection strain for all constructs. Animals were cultured at 20°C on NGM plates with *E. coli* (OP50) according to standard condition (S. Brenner, 1974). Fluorescent tags were inserted at the 3’ end of *pgl-1* endogenous locus by CRISPR genome editing using the self-excising cassette as described (Dickinson et al., 2015). To create the 5’ and 3’ *pgl-1* homology arms we used the following primers:

pgl-1 5’ arm F: GGAGAAGTGTTGTTTGTCCG

pgl-1 5’ arm R: GAAACCTCCACGGCCTCCCCGACCCCCGTAACC

pgl-1 3’ arm F: TAAACTCCAACTATTGAATGTTTAATTTG

pgl-1 3’ arm R: GGCCTCCCTATTAGACTTGC

Silent mutations were included in ‘pgl-1 5’ arm R’ at the site of guide RNA targeting to protect the plasmid repair template from cleavage. Homology arms also contained 20-30bp sequences specific to the mTagBFP2 or mKate2 vector for amplification from N2 genomic DNA and subsequent cloning into digested pDD287 (Addgene #70685) for mKate2 or pJJR81 (Addgene #75029) for mTagBFP2 vectors via isothermal cloning (Gibson et al., 2009). Correct sequences of constructed plasmids were confirmed with Sanger sequencing.

The guide RNA for *pgl-1* was generated by ligating oligos containing the guide sequence into BsaI-digested pRB1017 (Addgene #59936) and are the following sequences for PGL-1: 5’-tcttGGGGGTCGTGGTGGACGCGG-3’ and 5’-aaacCCGCGTCCACCACGACCCCC-3’. Plasmids were microinjected in the following concentrations: 50ng/µL pJW1259 (eft-3::Cas9), 50ng/µL *pgl-1* sgRNA, 25ng/µL respective PGL-1 repair plasmid, and 2.5-10ng/µL GFP or mCherry co-injection markers. Transgenic animals were confirmed by PCR and are available from the Phillips lab by request.

**Microscopy:** Adult animals were standardized for age by selection of L4 worms on the day preceding imaging. Undissected animals were mounted and imaged in <1% sodium azide in M9 buffer solution to prevent movement. For 1,6-hexanediol experiments, dissected animals were imaged immediately after dissection in either M9 buffer alone or a solution of 5% 1,6-hexanediol dissolved in M9 buffer. At least 5 gonads were analyzed for each genotype. For heat shock experiments, plates of animals were wrapped in Parafilm and placed in a 34°C incubator for 3 hours. Animals were mounted and imaged in less than five minutes after removal from incubator. At least 5 animals were analyzed for each genotype. For transcriptional inhibitor experiments, adult animals were microinjected with 200µg/mL of α-amanitin until the solution flowed around the germline bend. A vehicle control injection of RNase free water did not disrupt foci. The observed results are reproducible despite slight variation in microinjected volume and gonad integrity. At least 4 animals were analyzed for each genotype. Due to injection and imaging time requirements, animals were imaged 5 hours ± 15minutes post-injection. All live imaging was performed on a DeltaVision Elite (GE Healthcare) microscope using a 60x N.A. 1.42 oil-immersion objective. Six 0.20µm Z-stacks were collected and compiled at maximum intensity projections to create each image. Images were pseudo-colored and brightness/contrast was increased for clarity using Adobe Photoshop.

## Reagents

DUP75 *pgl-1(sam33[pgl-1::gfp::3xFLAG]) IV*

USC1082*pgl-1(cmp145[pgl-1::mKate2::Lox2272::3xMyc]) IV*

USC1269*pgl-1(cmp226[pgl-1::mTagBFP2::loxP::3xFLAG]) IV*
